# The Nutraceutical Promise of *Phaseolus vulgaris* L.: Bioactive Compounds for Health Promotion and Prevention of Chronic Noncommunicable Diseases

**DOI:** 10.3390/biology15080659

**Published:** 2026-04-21

**Authors:** Clizia Bernardi, Federica Finetti, Lorenza Trabalzini

**Affiliations:** Department of Biotechnology, Chemistry and Pharmacy, University of Siena, via Aldo Moro 2, 53100 Siena, Italy; clizia.bernardi2@unisi.it (C.B.); lorenza.trabalzini@unisi.it (L.T.)

**Keywords:** *Phaseolus vulgaris* L., cancer, cancer prevention, colon cancer, breast cancer, chemical composition, cardiovascular diseases, diabetes

## Abstract

Dietary habits are pivotal in preventing chronic noncommunicable diseases, as vegetable-rich diets provide over 25,000 bioactive phytochemicals that modulate cell-signaling and metabolic pathways. Consequently, nutraceuticals and functional foods are increasingly recognized for their potential to prevent chronic pathologies. Among functional foods, *Phaseolus vulgaris* L. (common bean) stands out as a critical resource for global nutrition and disease prevention. Beyond its role in food security and environmental sustainability, the common bean offers extraordinary nutrient density, providing a unique “protein plus fiber” package and a source of health-promoting active ingredients. In this review, special emphasis is placed on the bean’s role in preventing or mitigating cardiovascular diseases and cancer, driven by bioactive molecules that modulate metabolic and cell-signaling pathways. Practical evidence of this growing interest is demonstrated by the surge in scientific literature over the last 50 years, as shown by PubMed and Scopus data. By synthesizing data from original research and existing reviews, this work highlights how incorporating common beans into the diet represents a strategic, health-conscious choice with potential therapeutic benefits for human health.

## Simple Summary

Dietary habits are essential in preventing long-term health conditions often linked to our environment and lifestyle. Eating plenty of vegetables is particularly effective because plants contain thousands of natural compounds that help regulate how our bodies function at a cellular level. This study focuses on the common bean, a widely available food with a remarkable nutritional profile. Beyond providing basic nutrients like protein, fiber, and vitamins, beans are rich in specific natural molecules that act as protective agents for the body. This review gathers existing scientific evidence to show how these molecules can help prevent the development of serious issues, specifically cardiovascular diseases and certain types of cancers. The findings suggest that including common beans in our daily meals is a simple and affordable way to protect our health. In this work we highlight how accessible dietary choices can significantly improve well-being and reduce the global burden of chronic illness, offering a natural strategy for a longer and healthier life.

## 1. Introduction

The genus *Phaseolus* is a group of plants within the family *Fabaceae* (the legume family) and includes approximately 70 species. Five of these species were domesticated: *P. vulgaris* (common bean), *P. lunatus* (lima bean), *P. acutifolius* (tepary bean), *P. coccineus* (scarlet runner bean), and *P. dumosus*/*P. polyanthus* (year bean) [1].

Among these, *Phaseolus vulgaris* L. is the most significant edible legume in the world for both direct consumption and production [2].

Common beans are annual plants exhibiting extensive variation in growth, maturation rates, and environmental adaptation, with more than 40,000 varieties. They were domesticated approximately 8000 years ago in Mexico and Peru and are now cultivated in temperate and subtropical regions for their edible dry seeds, commonly known as navy, kidney, red, black, pinto, and cranberry beans [3].

Countries with the highest common bean consumption include Brazil (19.7%), India (19.7%), Mexico (7.7%), the United States (6.6%), Tanzania (2.7%), and Uganda (2.7%). In Southern Europe, the main producers are Greece, with an annual production of approximately 79,000 tons, followed by Spain and Italy, which produce around 12,000 tons and cultivate over 6411 hectares [3].

In the contemporary food landscape, the common bean is no longer considered a “poor man’s food” but is increasingly recognized as one of the most precious resources for the future of global nutrition. Scientific research and public health policies now agree in identifying legumes, and beans in particular, as a fundamental pillar for addressing the challenges of the 21st century, from combating malnutrition to the climate crisis [3,4].

The nutritional importance of beans lies in their extraordinary nutrient density. They represent a primary source of vegetable proteins, slow-release carbohydrates, and dietary fibers. However, their true value lies in their accessibility. Unlike animal protein sources, which are unsustainable due to the requirement of expensive cold chains and intensive production processes, beans are easy to store, have a long shelf life, and have low production costs [5,6]. This makes them a democratic health tool, capable of guaranteeing a high-quality protein intake even for populations with scarce economic resources.

The contribution of beans to global food security is twofold: on the one hand, they help combat undernutrition; on the other, they contribute to the prevention of numerous diseases. In fact, they meet all the minimum dietary requirements recommended by the World Health Organization (WHO) and the Food and Agriculture Organization (FAO) and are rich in various nutrients and phytochemicals [7,8]. In developing countries, the introduction of biofortified varieties (naturally enriched with iron and zinc through breeding) contributes to combatting anemia and micronutrient deficiencies [9]. In Western societies, the consumption of beans helps, for example, to counteract obesity, type 2 diabetes, and cardiovascular diseases, thanks to their low glycemic index and total absence of cholesterol [6].

Beyond their nutritional profile, beans are a natural leader in sustainability. *P. vulgaris* plants possess the symbiotic capacity to fix atmospheric nitrogen in the soil, thereby significantly reducing the need for synthetic fertilizers. Furthermore, the production of protein from beans requires only a fraction of the water and land required for beef or pork production, making beans a preferred choice for a food system that respects planetary boundaries [5,10].

Beans are not simply a meat substitute, but an alternative that offers additional advantages. While meat provides proteins often accompanied by saturated fats, beans offer a “protein plus fiber” package that supports the health of the gut microbiota [11]. Although they contain limiting amino acids (such as methionine), a simple combination with cereals can provide a complete amino acid profile [12], comparable to that of animal sources but with a significantly lower environmental and inflammatory impact. Repositioning the bean at the center of the human diet is not only a health-conscious choice, but an act of sustainability. Integrating this food into dietary patterns promotes a consumption model that combines agronomic efficiency with nutritional excellence [13].

**Figure 1:**
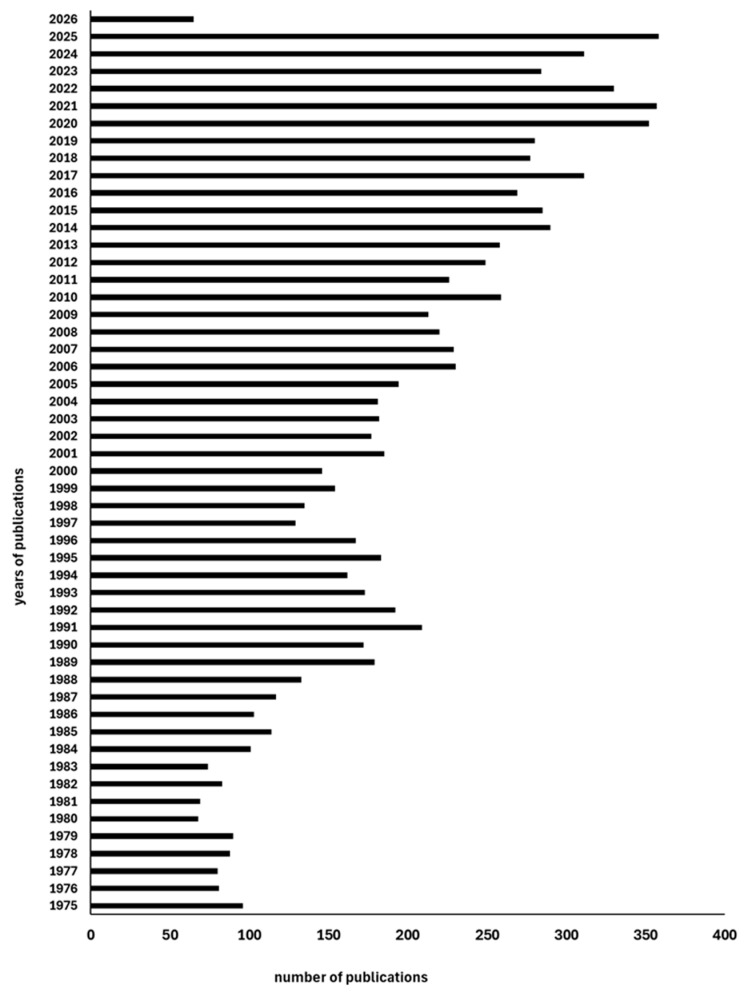
Annual number of publications on *Phaseolus vulgaris* from 1975 to 2026.

For these reasons, attention toward beans has increased considerably in recent years, driven by their high nutritional value and the functional properties of common beans [14]. Practical evidence of this growing interest is also provided by the large number of scientific publications in which beans are investigated from agronomic, environmental sustainability, and biodiversity perspectives, as well as from nutritional and nutraceutical viewpoints. Figure 1 illustrates the increasing number of publications over the last 50 years, based on a PubMed search using “*Phaseolus vulgaris*” as the keyword.

Within this context, the present review aims to emphasize the health benefits of bean consumption, with a specific focus on their role in preventing or mitigating cardiovascular diseases and cancer. A comprehensive literature search was performed using PubMed and Scopus, employing keywords such as “*Phaseolus vulgaris*”, “*Phaseolus vulgaris* and cancer”, and “*Phaseolus vulgaris* and cardiovascular disease”. Both existing reviews and original, more specialized scientific articles were considered.

## 2. Nutritional and Functional Components of *P. vulgaris*

Common beans contain several key functional ingredients and are a rich source of proteins, carbohydrates, unsaturated fatty acids, dietary fiber, vitamins, and minerals.

The protein content ranges from 16% to 33%, depending on the bean variety, environmental and cropping conditions, year of production and geographical location. Climate, soil characteristics, and fertilization practices also influence protein levels [15]. 

Compared with other legumes, common beans have a higher glutelin content (20–30%) which is primarily concentrated in the endosperm. The major protein fractions (50–70%) are globulins and albumins [15]. Furthermore, beans are rich in essential amino acids, including lysine, making them a good complement to cereal proteins, which are typically deficient in this amino acid. In addition, phaseolin, a glycoprotein containing neutral sugars, mainly mannose, accounts for approximately 40–50% of the total seed protein content [16].

Carbohydrates are the main component of common beans, accounting for approximately 55–65% of their dry weight. Among these, polysaccharide starch is the predominant fraction [17,18] while mono-, di-, and oligosaccharides occur only in trace amounts [2]. Bean starch can be hydrolyzed into oligodextrin and glucose by different enzymes such as α- and β-amylases [16]. The starch, dietary fiber, and non-starch polysaccharides present in beans—such as pectins, gums, hemicelluloses, inulin, fructans, stachyose, and raffinose—serve as fermentable substrates for the gut microbiota. This fermentation supports intestinal health and contributes to beneficial physiological effects [17].

The lipid fraction, which represents the smallest proportion among the macronutrients (1.5–6.2 g/100 g), is composed of various acylglycerides, with mono- and polyunsaturated fatty acids as the predominant types [19]. Polyunsaturated fatty acids, particularly omega-3 (alpha-linolenic acid) and omega-6 (linoleic acid), account for approximately 61% of the total fatty acids in common beans, with linolenic acid being the most abundant polyunsaturated fatty acid. These fatty acids confer several health benefits, including supporting cardiovascular function, enhancing the immune response, and preventing obesity and dyslipidemia [17,20]. They also play important roles in tissue development and overall physiological processes. In addition, oleic and linoleic acids are present in beans in variable concentrations, ranging from 7.8 to 13.8% and 16.7 to 25.8%, respectively [16].

Common beans are also rich in vitamins and minerals [21,22]. Soluble vitamins include thiamine, riboflavin, niacin, folic acid, and vitamins A, B6, C, E, and K, contributing to bone health and antioxidative effects [17]. The predominant cations found in common beans are calcium, magnesium, and potassium, with calcium exhibiting greater bioavailability than potassium or magnesium [2]. Furthermore, beans contain essential elements such as selenium, iron, and zinc, which play crucial roles in immune function and may help reduce the risk of cancer [16].

However, despite their high nutritional value, the bioavailability of several nutrients in beans can be influenced by the presence of antinutritional compounds. Bean seeds contain various substances such as lectins, phytic acid, condensed tannins, raffinose-family oligosaccharides, polyphenols, and saponins, which may exert both beneficial biological activities and antinutritional effects [23,24,25]. In particular, phytic acid can strongly chelate divalent and trivalent minerals such as iron and zinc, thereby reducing their bioavailability, while polyphenols and tannins—mainly located in the seed coat—may inhibit protein digestibility and mineral absorption [26,27]. Lectins, such as phytohaemagglutinins, can also interfere with intestinal integrity if beans are consumed without adequate processing, as these proteins are capable of binding to epithelial cells of the gastrointestinal tract and impairing nutrient absorption [28].

The bioavailability and physiological effects of bean nutrients may therefore vary depending on dietary patterns, intake levels, and food preparation methods. Regular consumption of beans within balanced dietary frameworks, such as the Mediterranean or other plant-based diets, can enhance nutrient intake and promote gut and metabolic health [29,30]. Epidemiological studies suggest that consuming at least two servings of legumes per week provide significant protection against metabolic and cardiovascular disorders [31,32,33]. Therefore, recommending realistic and frequent serving sizes is crucial for effectively translating the nutraceutical potential of beans into tangible health benefits. Culinary preparation techniques also influence both the digestibility and the content of bioactive compounds. Soaking and prolonged cooking reduce anti-nutritional factors (such as phytates and lectins), thereby improving mineral and protein absorption. Conversely, excessive cooking or discarding cooking water may reduce certain beneficial compounds [34,35,36].

## 3. Phytochemical Composition of *P. vulgaris*

In addition to being a significant source of protein, carbohydrates, fiber, and minerals, common beans are rich in phytochemicals that contribute to their health-promoting properties. These compounds include phenolic acids (chlorogenic, syringic, and caffeic acids), flavonoids (kaempferol, pelargonidin, cyanidin, delphinidin), flavan-3-ols, anthocyanins, condensed tannins, and saponins [37,38,39,40,41].

Each class is characterised by distinct chemical structures that determine their redox behaviour, bioavailability, and molecular targets. Increasing evidence indicates that these phytochemical classes exert complementary and synergistic effects by modulating oxidative stress, inflammatory signalling, metabolic pathways, and cellular homeostasis, thereby contributing to the prevention of chronic diseases such as cancer, cardiovascular disorders, and neurodegenerative conditions [42].

### 3.1. Phenolic Compounds

Phenolic compounds are secondary metabolites with different structures and functions, characterized by the presence of an aromatic ring bearing one or more hydroxyl groups [43]. They are commonly classified according to the number and configuration of phenolic hydroxyl groups and aromatic ring systems within their molecular structures. This classification gives rise to several principal subclasses, including simple phenols, phenolic acids, flavonoids, lignans, stilbenes, and tannins (Table 1).

The predominant phenolic acids in both raw and cooked beans include gallic, vanillic, *p*-coumaric, ferulic, sinapic, and chlorogenic acids [44].

**Table 1:** Major bioactive compounds and their reported activities.

Classes	Compounds	General Activities and Properties
**Phenolic Acids** 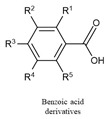 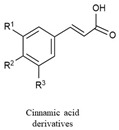	**Benzoic acid derivatives** *p*-Hydroxybenzoic acid [14,42] Gallic acid [14,39,45,46,47,48,49,50] Vanillic acid [14,47,49] **Cinnamic acid derivatives** Chlorogenic acid [14,46,47,48,49,50,51,52] *p*-Coumaric acid [39,46,47,49,53,54] *o*-Coumaric acid [43] Ferulic acid [14,39,46,47,50,51,52,54] Caffeic acid [14,46,47,49] Sinapic acid [39,54] Ellagic acid [53]	Neuroprotective, antioxidant, bacteriostatic, anti-melanoma properties, benefits in Parkinson’s and Alzheimer diseases [55,56,57,58,59,60,61,62,63,64,65].
**Flavonoids** 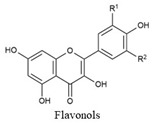 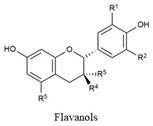 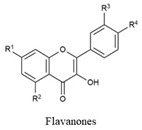 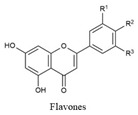 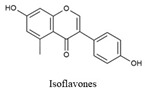	**Flavonols** Quercetin [39,46,49,50,52] Myricetin [14,46,51,53] Kaempferol [39,46,49,51,66] Rutin [14,49,50,52] **Flavanols** Catechin [14,39,46,49,50,51,52,53,54,67] Epicatechin [14,39,49,50,51,52] **Flavanones** Narigenin [39] Hesperetin [39] Naringin [39] **Flavones** Luteolin [14,50] Apigenin [14] **Isoflavones** Daidzein [14,39,51] Genistein [14,39] Biochanin A [39]	Antioxidant, anti-inflammatory, anticancer, cardioprotective, neuroprotective, antidiabetic [68,69,70,71,72,73,74,75,76,77,78,79,80,81].
**Stilbenes** 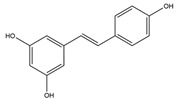	Resveratrol [39]	Anticancer, cardioprotective, neuroprotective, antimicrobial, anti-inflammatory, antidiabetic [82,83,84,85,86,87,88,89,90].
**Saponins** 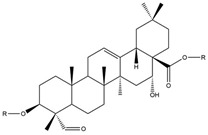	Phaseoside I, Soyasaponins [91,92,93]	Hypocholesterolemic, immunostimulant, anticancer, hypoglycemic, antithrombotic, diuretic, anti-inflammatory, cardioprotective, antioxidant, antiviral [94,95,96,97,98,99,100,101,102,103,104,105,106,107,108].
**Condensed tannins** 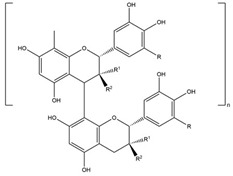	Proanthocyanidins (Proanthocyanidin dimer, Procyanidin B1 dimers, Procyanidin C1 trimer) [39,51,67,109]	Antioxidant, anti-inflammatory, anti-carcinogenic, anti-mutagenic and free radical scavenger properties, benefits in cardiovascular diseases and gut microbiome health [110,111,112,113,114,115,116,117,118,119,120].
**Anthocyanins** 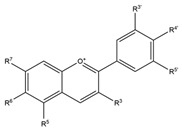	Delphinidin [14,48,49,51] Petunidin [14,39] Cyanidin [39,48,49,52,67] Malvidin [14,39,51] Pelargonidin [39,67]	Prevention of cancer, cardiovascular diseases, atherosclerosis, inflammation, oxidative stress [121,122,123,124,125].

Flavonoids are mainly found in the seed coat, whereas the cotyledon primarily contains non-flavonoid phenolic compounds, such as hydroxybenzoic and hydroxycinnamic acids [41]. Research on Mexican bean varieties has demonstrated that the seed coat, which represents approximately 11% of the total seed weight, contains the highest concentration of polyphenols. For example, the Flor de Mayo FM-38 variety contains 145 mg/g of phenolic compounds. In wild beans, total phenolic content ranges from 0.9 to 2.1 mg gallic acid equivalents (GAE) per gram, values comparable to those found in *Vaccinium* berries [45,126].

Thermal processing significantly reduces phenolic content. Cooking and frying decrease phenolic levels by approximately 50% and 64%, respectively, although these effects vary among genotypes. Dark-colored bean varieties generally have higher phenolic concentrations than lighter-colored ones, as reported in studies comparing Mexican and Turkish bean varieties [127]. Storage conditions also impact phenolic content and antioxidant activity. Elevated temperature and humidity cause significant reductions, while cooler, low-humidity environments help preserve these compounds [128]. Additionally, genotype, growing location, and environmental factors, such as irrigation and hydric stress, influence phenolic levels. For example, beans from the Jalisco race grown under specific conditions have higher phenolic contents than those from the Durango and Nueva Granada races [129].

As highlighted in Table 2, the reported values of total phenolic and flavonoid contents show a high degree of variability, largely depending on bean color, anatomical fraction, and extraction conditions. In particular, seed coats consistently exhibit markedly higher concentrations of phenolic compounds compared to cotyledons and whole seeds, confirming their role as the primary reservoir of flavonoids, tannins, and anthocyanins. Moreover, dark-colored beans generally present higher phenolic levels than light-colored varieties, while processing treatments such as cooking and digestion tend to reduce extractable compounds, although in some cases increasing their bioaccessible fraction.

#### 3.1.1. Phenolic Acids

Phenolic acids are key precursors for the synthesis of more complex phenolic compounds. They are classified into two main types: benzoic acid derivatives, such as *p*-hydroxybenzoic, vanillic, and gallic acids, and cinnamic acid derivatives, including ferulic, *p*-coumaric, and caffeic acids [144].

Both raw and cooked common beans contain significant levels of gallic, vanillic, coumaric, sinapic, ferulic, and chlorogenic acids [127]. In a study by Espinosa-Alonso et al., ferulic acid was identified as the predominant phenolic acid in 62 wild-type Mexican bean lines [45].

The impact of cooking on phenolic acids varies among studies. Some investigations reported no significant changes, whereas others found reductions in specific acids, including *p*-hydroxybenzoic, vanillic, coumaric, and ferulic acids, following cooking [81].

Phenolic acids provide diverse health benefits, particularly in neuroprotection, antioxidant defense, and disease prevention. Gallic acid exhibits antioxidant, bacteriostatic, and anti-melanoma activities and shows potential as a therapeutic agent for brain tumors [57]. Chlorogenic acid (CGA) protects against beta-amyloid peptide-induced apoptosis, and possesses anti-amnesic, anticholinesterase, and radical-scavenging properties [56,58,145]. Moreover, CGA reduces brain damage and improves cognitive function in neonatal rats after hypoxia-ischemia by activating the Sirtuin 1 (Sirt1)/Nuclear factor erythroid 2-related factor 2 (Nrf2)–Nuclear factor-κappa B (NF-κB) signaling pathway, thereby reducing inflammation and oxidative stress [59]. Ferulic acid exhibits antioxidant, anti-inflammatory, and immunostimulant activities, improves demyelination, motor incoordination, and cognitive dysfunction in experimental models of multiple sclerosis, and promotes beta-amyloid degradation [60,61,62]. Caffeic acid has neuroprotective activity, inhibits calcium efflux and Tau phosphorylation, protects neurons from oxidative stress, and, together with coumaric acid, shows potential benefits in Parkinson’s disease [63,64,65]. In addition, caffeic acid has been shown to protect against cerebral ischemia–reperfusion injury in rats, likely via the inhibition of 5-lipoxygenase and NF-κB signaling [55].

#### 3.1.2. Flavonoids

Flavonoids share a common structure consisting of two aromatic rings linked by a three-carbon bridge that forms an oxygenated heterocyclic ring. They are classified into six subclasses: flavonols, flavones, isoflavones, flavanones, anthocyanidins, and flavanols. Key flavonoids present in raw and cooked beans include catechin, kaempferol, quercetin, myricetin, and procyanidin [44] (Table 1).

The health benefits of flavonoids are mainly due to their antioxidant activity, modulation of detoxifying enzymes, and inhibition of cell proliferation. Flavonoids have been linked to a reduced risk of cancer, cardiovascular diseases, type 2 diabetes, and neurodegenerative disorders such as Alzheimer’s and Parkinson’s diseases [93]. They also exert anti-allergic effects by modulating platelet aggregation and inducing smooth muscle relaxation [146].

Furthermore, compounds such as resveratrol and procyanidin B1 have been reported to enhance longevity and brain function [69]. Quercetin exhibits multiple biological activities, including anti-inflammatory, immunomodulatory, anticancer, and antiviral effects, as well as reduction in lipid peroxidation, platelet aggregation, and capillary permeability. In vitro studies show that quercetin suppresses lipopolysaccharide (LPS)-induced tumor necrosis factor alfa (TNF-α) and Interleukin-8 (IL-8) production in macrophages and lung A549 cells, respectively [70,71]. Quercetin also inhibits inflammation-related enzymes, such as cyclooxygenase (COX) and lipoxygenase (LOX), and blocks proto-oncogene tyrosine-protein kinase Src (Src) and spleen tyrosine kinase (Syk)-mediated signaling by limiting the Toll-like receptor 4 (TLR4)/myeloid differentiation primary response 88 (MyD88)/phosphoinositide 3-kinase (PI3K) complex formation and downstream inflammatory pathways [72,73]. Moreover, quercetin mitigates hydrogen peroxide (H_2_O_2_)-induced inflammation in endothelial cells by downregulating vascular cell adhesion molecule 1 (VCAM-1) and cluster of differentiation 80 (CD80) expression [74].

In animal models, quercetin reduces TNF-α and nitric oxide (NO) production in visceral adipose tissue and downregulates nitric oxide synthase (NOS) expression in obese Zucker rats [56,75]. It also suppresses leukocyte recruitment, lowers chemokine levels and lipid peroxidation markers, and enhances antioxidant enzyme activity [76]. In experimental autoimmune myocarditis (EAM), quercetin modulates pro-inflammatory (TNF-α, IL-17) and anti-inflammatory (IL-10) cytokines [77]. In diet-induced obesity, quercetin suppresses oxidative stress, NF-κB activity, and immune cell activation while enhancing mitochondrial oxidative phosphorylation [78].

Epidemiological studies have associated flavonoid consumption with decreased disease risk. Flavonols reduce the incidence of type 2 diabetes, while anthocyanidins and flavan-3-ols reduce cardiovascular disease mortality [79]. Mexican black bean varieties, such as Negro San Luis, are particularly rich in flavonoids, including quercetin 4-O-galactoside and kaempferol 3-O-glucoside. Studies have indicated that quercetin content is highest in black beans, whereas kaempferol is abundant in bayo beans [127].

Isoflavonoids, a subclass of flavonoids, act as phytoestrogens owing to their structural and functional similarity to 17 β-estradiol, allowing them to interact with estrogen receptors. Isoflavone content varies among plant species and genotypes and is affected by environmental factors, including crop location, temperature, fertilization, pests, and processing methods. Although common beans are generally not considered significant sources of isoflavones, some studies have identified these compounds in specific varieties. For example, Brazilian black beans show higher isoflavonoid levels, including daidzein [147].

Isoflavones, especially genistein, demonstrate anticancer activities by inhibiting both hormone-dependent and independent cancer cells growth (e.g., breast, prostate, colon, skin) with IC_50_ (Half maximal inhibitory concentration) values of 5–40 μM [80]. Genistein also suppresses metastasis, blocks protein tyrosine kinases and DNA topoisomerases, and interferes with cell signaling pathways [81]. In addition, isoflavones exhibit antioxidant activity, which further contributes to their health benefits [81].

#### 3.1.3. Anthocyanins

Anthocyanins, a subclass of flavonoids, are responsible for red, black, and pink pigmentation in bean seed coats. Their content varies according to the bean variety and seed coat color, with darker beans containing higher levels than lighter beans [127].

Compared with other anthocyanin-rich foods such as berries, which may contain up to 366 mg/100 g [148], beans generally contain lower amounts. In raw beans, anthocyanin levels range from 0.01 to 1.85 mg cyanidin-3-glucoside equivalents (C3G) per gram (g) [45,149,150]. However, black beans show higher levels, with reported values between 1.94 and 3.47 mg C3G/g [151], confirming the strong association between seed coat pigmentation and anthocyanin content.

Anthocyanins possess strong antioxidant properties and are associated with multiple health benefits, including the prevention of cancer, atherosclerosis, and inflammation [122].

Anthocyanins extracted from black soybeans have been shown to exert anti-inflammatory and antifibrotic effects on penile plaque formation in rat models of Peyronie’s disease (PD) [123].

Zheng et al. demonstrated that anthocyanins and carotenoids may help prevent cardiovascular diseases through activation of the serine/threonine protein kinase B (Akt)/Nrf2 pathway, suggesting that combined dietary intake of these phytochemicals may provide protect against oxidative stress [124]. Similarly, Zhang et al. showed that anthocyanins inhibited TNF-α-induced IL-8 and TNF-α secretion in Caco-2 cells by blocking NF-κB and mitogen-activated protein kinase (MAPK) pathways, indicating a role in mitigating inflammation-related diseases [125].

Anthocyanins also reduced cell viability in colon cancer HT-29 cells and decreased pro-inflammatory markers (TNF-α, IL-1β, IL-6) in both HT-29 and non-malignant CCD-18Co colon cells. In addition, they reduced TNF-α-induced reactive oxygen species (ROS) production by 17.3% in CCD-18Co cells [121].

Despite these benefits, no universally accepted dietary reference intake for anthocyanins exists. China proposes a daily intake of 50 mg, and the FAO/WHO recommends 2.5 mg/kg/day for anthocyanins derived from grape skin extracts. However, global guidelines remain under development due to insufficient toxicological data [152].

#### 3.1.4. Condensed Tannins

Tannins are bioactive secondary metabolites in plants [153,154]. In common beans, tannins are primarily concentrated in the seed coat rather than the cotyledons and, together with anthocyanins, they determinate the color of the bean seed coat.

Condensed tannins, also known as proanthocyanidins, are flavonoid polymers that bind proteins, metals, and polysaccharides to form complexes. Their concentrations are closely associated with seed coat color, as reported in several Mexican bean varieties [45,111].

Condensed tannins exhibit antioxidant, anticarcinogenic, antimutagenic, and free radical scavenger properties [111].

Proanthocyanidins inhibit colon tumor growth by reducing vascular endothelial growth factor (VEGF) and angiopoietin 1 (Ang1) expression through reactive ROS scavenging [112,113]. They also suppress angiogenesis by downregulating matrix metalloproteinases 2 and 9 (MMP-2 and MMP-9), as well as VEGF, and Ang1 signaling [114].

Oligomeric procyanidin B2 inhibits prostate cancer cell growth by displacing testosterone from membrane androgen receptors, thereby reducing androgen signaling in both androgen-sensitive and -resistant prostate cancer cells [115]. In addition, proanthocyanidin B2 exhibits cytotoxic effects against human breast cancer cells (MCF-7) and lung fibroblasts, although with lower potency than paclitaxel [116].

Another condensed tannin compound, Weimaining (WMN), inhibits breast cancer growth in mice by enhancing apoptosis and increasing caspase-3 expression [117].

Tannins also help prevent cardiovascular diseases by neutralizing free oxygen and reactive nitrogen species, thereby reducing oxidative stress on low-density lipoprotein (LDL) cholesterol. They also improve insulin resistance, inhibit platelet aggregation and blood clot formation, suppress NO production, and exert anti-inflammatory activity [118,119,120].

Furthermore, tannins influence gut microbiota composition. In female rats, dietary tannins increased the abundance of *Bacteroidetes* and decreased *Firmicutes*, suggesting a role in modulating the microbiome profile [110].

### 3.2. Saponins

Common beans contain small amounts of saponins, bioactive compounds characterized by a hydrophobic steroidal or triterpenoid nucleus linked to hydrophilic sugar chains. Saponins are classified into groups A, B, and E based on aglycone structures, with variations in glycosylation and functional groups [68]. In common beans, the predominant saponins are soyasaponin A, soyasaponin B, and phaseoside I, with soyasaponin I being the most abundant [91]. Epidemiological and experimental studies highlight health benefits of saponins, including hypocholesterolemic, immunostimulant, anticancer, hypoglycemic, antithrombotic, diuretic, and anti-inflammatory effects [95,96,97,98,99]. Saponins also reduce the risk of cardiovascular disease, exhibit strong antioxidant activity, and may inhibit human immunodeficiency virus (HIV) infectivity in vitro [100].

Both in vitro and in vivo studies have shown that saponins exert antitumor effects through multiple mechanisms. They inhibit NF-κB activity, induce apoptosis, and suppress proliferation in colon cancer cells [101,155].

In melanoma models, saponins decrease cell proliferation by downregulating histone deacetylase 3 (HDAC3) and increasing p53 acetylation. These effects result in cell cycle arrest and tumor growth inhibition both in vitro and in vivo [106]. Combined treatments with agents such as sodium selenite further boost growth inhibition and apoptosis and reduce oxidative stress and autophagy [107].

Saponins also exhibit cardioprotective effects by mitigating anthracycline-induced toxicity and overcoming multidrug-resistance (MDR), thereby improving therapeutic outcomes without compromising cardiac safety [108]. Moreover, in non-small-cell lung carcinoma (H460), saponins reduced tumor size and weight by approximately 30%, showing efficacy comparable to the chemotherapeutic drug doxorubicin [94].

## 4. Antitumoral Potential of *P. vulgaris*

The validation of natural compounds follows a rigorous translational pathway, mirroring the stringent protocols required for synthetic pharmaceuticals. This process begins with robust preclinical investigations, including in vitro assays and in vivo animal models, to elucidate the underlying molecular mechanisms and safety profiles. However, preclinical success must be substantiated by well-designed clinical trials to confirm efficacy and bioavailability in humans.

**Figure 2:**
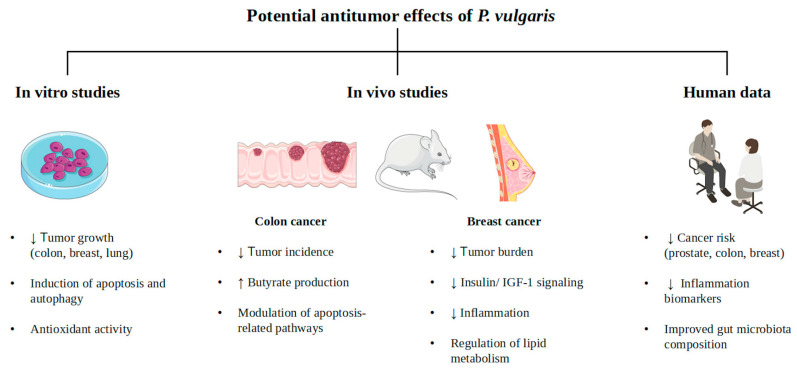
Summary of the potential antitumoral effects of *P. vulgaris* based on in vitro, in vivo, and human studies, highlighting antioxidant activity, modulation of cancer-related pathways, and effects on tumor growth, metabolism, and gut microbiota.↑ increasing; ↓ reduction.

In the specific context of nutraceutical science, where bioactive compounds are often consumed as part of a complex diet, the integration of rigorous epidemiological studies is indispensable. Large-scale observational cohorts and meta-analyses provide essential evidence regarding long-term exposure and population-wide health outcomes, effectively bridging the gap between controlled clinical settings and real-world nutritional impact. The antitumor potential of common beans has been demonstrated in several experimental models. In vitro studies highlight bioactive beans compounds, particularly polyphenols and proteins, for their antioxidant, anti-inflammatory, and pro-apoptotic properties, respectively. Animal studies on colon and breast cancer models show that bean-based diets reduce tumor incidence and promote apoptosis. Moreover, although epidemiological data in humans suggest a correlation between high bean consumption and a reduced risk of certain cancers, particularly colorectal, prostate, and breast cancers, further clinical research is required to confirm these protective effects (Figure 2).

### 4.1. In Vitro Studies

The initial step in determining the specific biological activity of a natural compound or extract involves in vitro testing using cellular models that accurately represent the disease of interest. In this context, numerous studies have evaluated the potential anticancer properties of various bean extracts. Such preliminary data can therefore provide a rationale for further investigations in animal models.

Several studies report that common beans exert pro-apoptotic, anti-inflammatory and antiproliferative effects on various cancer cell types using different bean extracts [53,156,157,158,159].

Ombra et al. [53] analyzed the phenolic profiles of 12 *P. vulgaris* ecotypes and identified substantial variability in phenolic composition. Antioxidant activity, assessed using the 2,2-diphenyl-1-picrylhydrazyl (DPPH) radical assay, showed strong inhibition capacity, particularly in non-pigmented samples. Extracts also inhibited proliferation of Caco-2, MCF-7, and A549 cells, with only marginal differences in antioxidant activity after cooking [53].

A newly characterized *P. vulgaris* variety, Fagiola di Venanzio, was rich in polyphenols and exhibited significant antioxidant, anti-inflammatory, and antiproliferative activities in colon cancer models [156,158]. Moreover, the bioaccessible fraction of several white bean varieties obtained after *in vitro* gastrointestinal digestion of cooked beans promoted colon cancer cell death through autophagy induction [159].

Two common bean varieties, Bayo Victoria and Negro 8025, were evaluated for cytotoxic effects against HT-29 cells. Bayo Victoria showed greater diversity in phenolic compounds, whereas Negro 8025 exhibited higher total phenolic content and stronger pro-apoptotic activity. Both cultivars modulated proteins involved in cell-cycle arrest and apoptotic pathways, underscoring their potential role in cancer prevention and overall health promotion [157].

Red kidney bean coat extracts also exhibited potent anti-melanoma activity. The *n*-BuOH fraction was identified as the most effective, inducing apoptosis and vacuolization in melanoma cells. Transcriptomic analysis suggested that apoptosis was regulated through the PI3K-Akt–Forkhead box O (FOXO) and Mouse double minute 2 homolog (MDM2)–p53 signaling pathways, with quercetin identifies as a key regulatory compound [160].

Collectively, these studies support the hypothesis that common beans, particularly those rich in bioactive polyphenols, may contribute to cancer prevention and the promotion of human health.

### 4.2. In Vivo Studies

Building on the promising anticancer activity observed in vitro, the efficacy of *P. vulgaris* extracts was subsequently evaluated in vivo using animal models. Specifically, we present several examples of chemically induced colon and breast tumors. The data illustrated below indicate that dietary supplementation with common beans can effectively attenuate carcinogenic processes.

A study involving 53 five-week-old male F344 rats investigated the effects of diet on the development of colon cancer. The rats were divided into three groups: control (11 rats), casein diet (21 rats), and bean diet (21 rats) groups. Both the casein and bean diet groups were exposed to the carcinogen azoxymethane (AOM) once a week for two weeks, whereas the control group received the casein diet without AOM exposure. All diets were isocaloric, with protein content adjusted to 18 g/100 g (casein-based) and fat content to 5 g/100 g (corn oil). The bean diet consisted of dry cooked beans incorporated into pelleted experimental diets. Rats fed the bean diet developed significantly fewer colon adenocarcinomas than those on the casein diet (5 vs. 22 tumors, *p* < 0.05). Moreover, fewer bean-fed rats developed colonic tumors (24% vs. 50%, *p* < 0.05), and tumor multiplicity was reduced in the bean group. Bean-fed rats had significantly fewer tumors per tumor-bearing rat (1.0 ± 0.0) than casein-fed rats (2.5 ± 0.6, *p* < 0.05). These findings suggest that dry cooked beans contain anticarcinogenic compounds capable of inhibiting AOM-induced colon cancer in rats [161].

Additional studies reported chemopreventive effects of bean-derived components in AOM-induced colon cancer. A polysaccharide extract (PE) from the *P. vulgaris* cultivar Negro 8025 increased the production of short-chain fatty acids (SCFA), particularly butyrate, reaching a concentration of 6.7 mmol/g in the cecum. PE administration reduced the number of aberrant crypt foci (ACF), precursors to colon cancer, and modulated gene expression by upregulating Bcl-2 associated X-protein (Bax) and caspase-3 (pro-apoptotic genes) and downregulating retinoblastoma protein (Rb). These findings suggest that butyrate produced from PE fermentation plays a significant role in colon cancer suppression [162].

Similarly, the non-digestible fraction (NDF) from the Bayo Madero cultivar and whole cooked beans (CB) significantly suppressed ACF formation. Rats fed CB or NDF showed ACF levels of 0.8 and 1.5 ACF/distal zone, respectively, compared to 6.6 ACF/distal zone in AOM-only rats. Both diets reduced β-glucuronidase activity in caecal, colonic, and faecal contents, indicating direct chemoprotective effects during early colon carcinogenesis [163].

Further work demonstrated that NDF from the Negro 8025 cultivar reduced early-stage colon carcinogenesis in male Sprague Dawley rats. Rats treated with NDF and AOM exhibited significantly fewer colonic ACF, increased G1 phase cell population (83.1%), and reduced cell proliferation compared to AOM-only. NDF also promotes apoptosis by inducing cell cycle arrest and morphological changes linked to programmed cell death [164].

García-Cordero et al. [165] showed that phaseolin, a protein isolated from common beans, exerted strong chemopreventive potential effect against AOM-induced damage in mice. Phaseolin inhibited lipid peroxidation, protein oxidation, and nitrite production by 100% and reduced DNA damage by over 90% at higher doses. It also decreased ACF formation by 84% and exhibited significant antioxidant activity, as assessed by the DPPH assay [165].

In a chemically induced breast cancer model in rats, high dietary bean intake (60% wt/wt) reduced carcinoma burden by 62.2% (*p* < 0.001), primarily through enhanced apoptosis. Bean consumption affects the phosphorylation of key proteins in the mammalian target of rapamycin (mTOR) signaling pathway, eukaryotic translation initiation factor 4E-binding protein 1 (4E-BP1), p70S6 kinase, AMP-activated protein kinase (AMPK), and Akt, even at lower dietary levels (7.5–30% *w*/*w*). Plasma insulin and insulin-like growth factor 1 (IGF-1) concentrations decreased by 36.3% and 38.9%, respectively (*p* < 0.001), alongside a 23% reduction in C-reactive protein, highlighting the role of mTOR pathway modulation in bean-mediated anticancer effects [166].

Using metabolomic analysis via liquid chromatography–time-of-flight mass spectrometry (LC-TOF-MS), Mensack et al. [167] distinguished the metabolomic profiles of normal mammary glands and mammary carcinoma tissues in control-fed versus bean-fed rats. Principal component analysis revealed differences in eicosanoid, fatty acid, triacylglycerol (TAG), and steroid metabolism. Variations in lipid metabolite levels mirrored the differential inhibitory effects of genetically distinct bean types on mammary carcinogenesis. In addition, bean consumption modulates pathways related to anti-cancer effects, including diacylglycerol-protein kinase C and eicosanoid-COX-2 regulation, as well as eicosanoid degradation mediated by 15-PG dehydrogenase [167].

### 4.3. Human Data

*P. vulgaris* has been associated with cancer prevention in several human observational studies [2,8], which collectively suggest an inverse relationship between bean consumption and the risk of multiple cancer types, including prostate cancer (approximately 22%) [168], colon cancer (up to 47%) [169] and breast cancer (approximately 67%) [170]. However, these estimates derive predominantly from epidemiological studies and vary across populations and study designs. Overall, epidemiological evidence can be considered moderate, showing consistent protective trends but substantial heterogeneity in effect size.

Colorectal carcinoma typically develops from neoplastic adenomatous polyps, with advanced adenomas representing a key step in colorectal cancer progression. Epidemiological studies investigating fruit and vegetable intake generally support a reduced risk of adenoma formation, although the specific contribution of legumes or common beans is less clearly defined. The Polyp Prevention Trial (PPT), a four-year multicenter randomized clinical trial, evaluated the effect of short-erm dry bean intervention on colorectal adenoma outcomes and reported a reduction in advanced adenoma recurrence; however, the findings did not reach definitive statistical significance, highlighting the limited strength of current interventional evidence [171].

Additional pilot studies have been conducted in colorectal cancer survivors to evaluate the impact of bean consumption on parameters that may affect the recurrence of colorectal cancer. These studies examined inflammatory biomarkers, glycemic index and gut microbiota composition. Sheflin et al. reported that navy beans consumption increases stool bacterial richness and diversity [172], while Baxter et al. reported that navy beans modulate the stool metabolome by affecting different metabolic pathways associated with colon health and reduced colorectal cancer risk [173]. Similarly, a diet enriched with navy beans and rice bran beneficially modulated host and gut microbial metabolism, modifying amino acids and lipid metabolites in plasma, urine and stool [174]. Moreover, consumption of navy beans alone promoted a metabolic shift characterized by increased plasma and urinary levels of metabolites with putative cancer-protective effects [175]. 

Despite these promising findings, human studies are limited by small sample sizes, short intervention durations, and the use of surrogate endpoints rather than clinical cancer outcomes, limiting the generalizability and causal interpretation of the results.

Taken together, current human evidence suggests a potential protective role of common bean consumption in cancer prevention, supported by reasonably consistent epidemiological associations across different populations. However, the overall level of evidence remains inconclusive, and well-designed, large-scale, long-term randomized clinical trials are still required to confirm efficacy, define dose–response relationships, and account for population-specific dietary patterns.

## 5. Other Biological Properties of *P. vulgaris*

**Figure 3:**
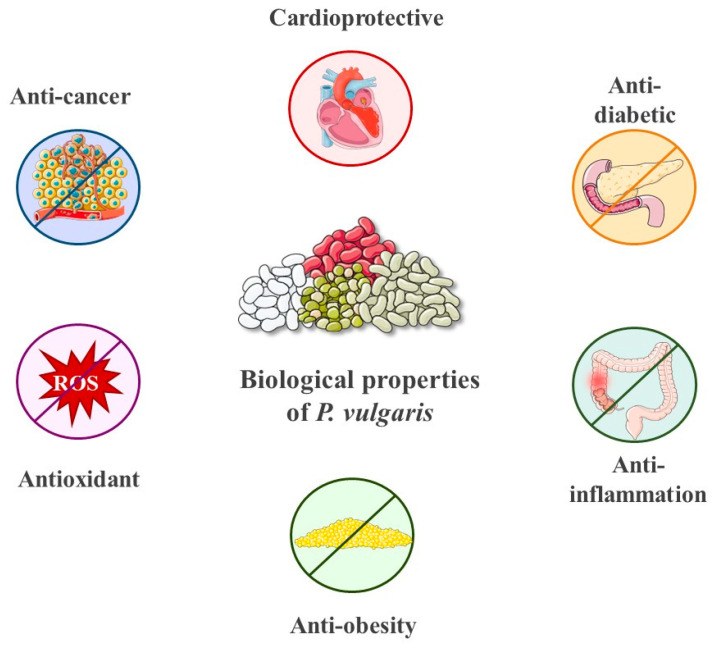
Biological activities of *P. vulgaris*, including its antioxidant, anti-inflammatory, antitumoral, cardioprotective, and metabolic regulatory effects, associated with human health benefits.

Thanks to its high content of proteins, dietary fiber, vitamins, minerals, and phytochemicals, including polyphenols, flavonoids, saponins, and resistant starch, *P. vulgaris* is not only a staple food worldwide but also a valuable source of bioactive compounds associated with a wide range of health benefits (Figure 3) [8].

### 5.1. Antioxidant and Anti-Inflammatory Activity

Dysfunction or imbalance in gut health can lead to chronic human diseases, including inflammatory bowel disease (IBD), obesity, colon cancer, diabetes, and neurological disorders. As discussed above, beans contain a variety of bioactive components, including fermentable non-digestible carbohydrates and phenolic compounds, which may contribute to their capacity to promote gut health [176]. The antioxidant properties of beans are largely attributed to the reducing capacity of polyphenols. These compounds scavenge and neutralize free radicals and inhibit lipid peroxidation [177].

Beyond their antioxidant potential, phenolic compounds exert a range of cellular effects, including anti-inflammatory activity. Bean-derived phenolic compounds modulate the NF-κB signaling pathway, suppressing the expression of pro-inflammatory mediators such as IL-6, TNF-α, and IL-1β [178,179].

Protein hydrolysates rich in bioactive peptides from Pinto Durango and Negro 8025 bean varieties, suppressed inflammation in macrophages by inhibiting Prostaglandin E2 (PGE-2) and NO production. They also inhibited NF-κB activation and the nuclear translocation of its p65 subunit [180].

Galdino Alves et al. [181] investigated how the postharvest storage duration (0, 3, and 6 months) influences the anti-inflammatory properties of Carioca, Madreperola, and Pontal beans. Cooked beans were subjected to simulated gastrointestinal digestion before being in human THP-1 macrophage-like cells. The storage period did not affect protein content, hydrolysis, hydropathicity, or antioxidant capacity. All hydrolysates reduced TNF-α levels by approximately 30%, while Madreperola hydrolysates significantly reduced IL-1β and PGE-2 levels. Bioactive peptides and phenolic compounds from Carioca beans reduced inflammation, and storage time did not affect their physicochemical or biological properties [181].

Black bean peel extracts exhibited strong inhibitory effects on COX-1 and COX-2, while aqueous extracts inhibited LOX, highlighting their potential against chronic inflammation [182]. Similarly, an aqueous extract from white beans exhibited in vitro antioxidant and anti-inflammatory activity by reducing IL-1β-induced ROS production, decreasing COX-2 expression and inhibiting the activation of NF-κB and Extracellular signal-related kinases 1 and 2 (ERK1/2) MAPK pathways [158].

Phaseolin, the major globulin found in bean seeds, has emerged as a promising therapeutic candidate for managing inflammation. It inhibits NO production and downregulates the expression of pro-inflammatory mediators such as COX-2, IL-1β, and TNF-α. Additionally, phaseolin inhibits macrophage adhesion by downregulating the adhesion molecule Ninj1 and reduces leukocyte recruitment in vivo. It also prevents NF-κB nuclear translocation [183].

In a mouse model, Adzuki bean consumption reduced lipid accumulation and inflammation caused by oxidative stress, effects attributed to their high content of anthocyanins, catechins, and saponins [184]. Similarly, in mice fed a high-fat diet, both bean protein hydrolysates and whole bean flour reduced markers of inflammation and oxidative stress, along with decreased expression of Peroxisome Proliferator-Activated Receptor-α (PPAR-α). These benefits were attributed to phenolic compounds (e.g., catechin, kaempferol) and bioactive peptides and proteins such as phytohemagglutinin, phaseolin and alpha-amylase inhibitors [185].

Furthermore, C57BL/6 mice fed a diet containing 20% navy bean or black bean flour exhibited a significant reduction in experimental colitis induced by dextran sodium sulfate. This diet decreased the expression of inflammation-related markers (IL-1β, TNF-α, Interferon gamma (IFN-γ), IL-17A, and IL-9), increased histological injury scores and apoptosis, thereby effectively mitigating symptoms of colitis and colon inflammation [176]. 

In summary, phaseolin and other bioactive compounds present in beans exhibit potent antioxidant and anti-inflammatory activities, underscoring their potential role in the prevention and management of chronic inflammation-related conditions.

### 5.2. Cardiovascular Activity

Bioactive compounds in *P. vulgaris*, including phenolics, kaempferol, and catechin, exhibit strong antioxidant and anti-inflammatory activities. These properties contribute to reduced lipid peroxidation, improved lipid profiles and overall vascular protection [186]. Additional cardioprotective components include omega-3 fatty acids, alpha-linolenic acid, conjugated linoleic acid, and phytonutrients such as folic acid, magnesium, tannins, sulfur amino acids, and phytoestrogens [17,187,188].

These cardioprotective effects are not limited to lipid regulation and oxidative balance but also extend to the modulation of platelet function. A methanolic extract of *P. vulgaris* showed inhibitory effects on platelet aggregation induced by adenosine 5′-diphosphate (ADP) and arachidonic acid, by suppressing Akt phosphorylation and reducing ATP secretion by up to 34% [189]. Bean lectins both stimulate platelet aggregation via the Src/Syk and PI3K/Bruton tyrosine kinase (BTK) pathways and reduce aggregation through NO production mediated by the eNOS/NO/cyclic guanosine monophosphate (cGMP)/protein kinase G (PKG) signaling pathway [190]. Other components, including glycine and arginine, modulate these effects by inhibiting calcium influx and enhancing NO production, respectively [191,192].

In addition to platelet modulation, beans and their hydrolysates support endothelial function by improving lipid metabolism, reducing inflammation, and upregulating endothelial nitric oxide synthase (eNOS) expression [193]. A low-molecular-weight peptide fraction (PV3) isolated from hardened beans enhanced endothelial cell viability and protected against H_2_O_2_-induced oxidative stress. PV3 increased NO release, reduced ROS levels, and promoted endothelium-dependent vasodilation in isolated aortic rings [194].

Beans also play a crucial role in mitigating metabolic syndrome (METS), a cluster of conditions including central obesity, insulin resistance, hypertension, glucose intolerance, and dyslipidemia, by contributing to dietary patterns rich in unsaturated fats, legumes, whole grains, fruits, vegetables, and nuts [195,196].

The α-amylase inhibitor (αAI) found in beans slows carbohydrate digestion and absorption, thereby reducing postprandial glucose spikes and lowering the glycemic index [197]. Clinical trials have shown that bean-derived αAI and alpha-glucosidase inhibitors promote weight loss and decrease triglycerides, cholesterol, and postprandial insulin levels by limiting starch assimilation and enhancing lipid and glucose metabolism [164]. Furthermore, bean hydrolysates and baked beans have shown to reduce body weight and lipid levels, while increasing antioxidant capacity in both clinical and animal studies [198]. Bean proteins also inhibit angiotensin-converting enzyme (ACE), contributing to blood pressure reduction [193].

Both clinical and experimental studies have demonstrated that beans improve vascular health by reducing LDL, total cholesterol, and hepatic triglycerides while enhancing overall lipid metabolism [199,200]. These effects are particularly relevant to atherosclerosis, a major contributor to cardiovascular disease (CVD), characterized by elevated LDL levels, LDL oxidation, and monocyte recruitment, ultimately leading to foam cell formation and plaque development [181,201]. Importantly, common beans improve lipid profiles without negatively affecting triglycerides, very-low-density lipoprotein (VLDL) cholesterol, or blood glucose levels [187]. Peptides from Carioca beans exhibited anti-atherosclerotic effects by inhibiting LOX-1, MMP-9, intercellular adhesion molecule 1 (ICAM-1), and pro-inflammatory cytokines, rivaling the efficacy of simvastatin [181].

Beans also contribute significantly to the prevention and management of diabetes. Venn et al. reported that regular consumption of dry beans supports glycemic control [202], and clinical studies have shown that three or more servings per week can reduce diabetes risk by approximately 35% [42]. In vitro studies confirm that beans inhibit α-amylase, α-glucosidase, and dipeptidyl peptidase-IV, contributing to their anti-hyperglycemic effects through phenolic compounds such as flavonoids, anthocyanins, catechin, myricetin 3-O-arabinoside [51,203]. Animal studies further demonstrate that these compounds lower blood glucose and glycated hemoglobin levels while increasing insulin concentrations [204]. Additionally, clinical studies have shown that regular intake of black beans over a three-months period significantly reduces plasma glucose and glycated hemoglobin levels, improving type-2 diabetes management through the presence of total phenolic compounds, tannins, and anthocyanins [205,206].

Epidemiological evidence consistently indicates that legume consumption, particularly beans, is inversely correlated with the risks of coronary heart disease, type 2 diabetes, and obesity [207,208]. Large population-based studies further support a 34% reduction in CVD incidence among individuals adhering to diets rich in beans or other low-glycemic foods, particularly in older adults [209].

Despite these well-documented health benefits, beans consumption has declined in recent decades, coinciding with the rising prevalence of chronic diseases such as obesity and CVD [127].

## 6. Conclusions

*P. vulgaris* beans are widely recognized for their nutritional value and potential health benefits, particularly in the prevention and management of several chronic non-communicable diseases including cancer, cardiovascular diseases, obesity, and diabetes. Although epidemiological data and clinical trials largely support the beneficial effects of bean consumption in cardiometabolic disorders, evidence regarding their role in cancer prevention remains more inconsistent. It is well established that the development of various cancer types, particularly colorectal cancer, is strongly influenced by environmental and dietary factors. Both in vitro and in vivo studies have shown that the non-digestible fraction of cooked beans can inhibit early stages of carcinogenesis by modulating apoptosis, cell cycle progression, inflammation, and DNA repair.

Furthermore, several experimental biases should be addressed when investigating the molecular mechanisms underlying the biological activity associated with bean consumption. In particular, a critical aspect in characterizing the biological activity of foods is the procedure used to prepare the extracts. Failure to mimic physiological conditions—especially by neglecting the digestive processes of the human gastrointestinal tract—may represent a significant limitation in understanding the true biological role of certain foods. To generate bean extracts that more accurately reflect human digestion, in vitro procedures simulating gastrointestinal conditions should be employed. Such approaches should account for digestive enzymes, pH variations, salt concentrations, and transit times characteristic of the different stages of the gastrointestinal tract.

Moreover, the biological activity of beans and other pulses may be attributed not only to their native chemical constituents but also to digestion-derived products, such as small peptides, which are not biologically inert. Indeed, the formation of potentially bioactive peptides following the digestion of beans and other pulses has been well documented [210,211]. In light of these considerations, more comprehensive and methodologically refined in vitro and in vivo studies are warranted.

Clinical evidence on the role of *P. vulgaris* in the prevention and treatment of colon cancer remains limited, underscoring the need for large-scale, well-designed human trials to clarify the relationship between bean consumption and cancer risk and to encourage further mechanistic investigations.

## Figures and Tables

**Table 2:** Bioactive compounds in common beans. * Values reported in original units as in the cited studies. TPC (Total polyphenol content): mg GAE/g (GAE, Gallic acid equivalent); TFC (Total flavonoid content): mg CE/g (CE, Catechin equivalent), mg QE/g (QE, Quercetin equivalent), mg RE/g (RE, Resveratrol equivalent); TCT (Total condensed tannins): mg CE/g (CE, Catechin equivalent), mg DE/g (DE, Delphinin equivalent); TAC (Total anthocyanin content): mg CE/g (CE, Catechin equivalent), mg C3GE/g (C3GE, Cyanidin-3-glucosie equivalent). nd: not detected. MAE: Microwave-Assisted extraction; UAE: Ultrasound-Assisted extraction; SCF: Supercritical fluid extraction.

Fraction	Extraction Method/Solvent	TPC [mg GAE/g]	TFC [mg CE/g or eq.] *	TCT [mg CE/g or eq.] *	TAC [mg C3GE/g or eq.] *	Ref
**White/light beans**						
Whole seed	MeOH 70%	~0.2–0.5	~0.03–0.1	~0.04–0.09		[130]
	MeOH 60% (0.3% HCl)	9.8–21.6		0.02–0.5		[131]
	Acetone 70% (0.5% Acetic acid)	0.7–1.0	1.3–1.5	1.5–2.3		[132]
Cotyledon	MAE Water	4.2–6.9				[133]
	MAE Ethanol 50–100%	1.0–8.7				[133]
	UAE Ethanol ~50%	~5.0–10.0	~2.0–5.0	nd		[134]
Seed coat	MAE Water	2.5–5.2				[133]
	MAE Ethanol 50–100%	1.1–8.3				[133]
	UAE Ethanol 42%/Ethyl acetate	~20.0–30.0	~5.0–10.0	nd		[134]
	MeOH 80%	1.5–20.1	0.3–15.2			[135]
Raw	Acetone	~0.8–1.2	~0.3–0.6		nd	[53]
Cooked	Acetone	~0.5–1.0	~0.2–0.5		nd	[53]
**Brown/Dark beans**						
Whole seed	MeOH 70%	~0.2–1.6	~0.04–0.8	~0.06–0.5		[132]
	MeOH 60% (0.3% HCl)	6.7–32.0		0.0–2.4		[131]
Cotyledon	MAE Water	4.0–7.2				[133]
	MAE Ethanol 50–100%	1.0–10.4				[133]
	Acetone 70% (0.5% Acetic acid)	2.3–2.4	0.3–0.4		nd	[136]
Seed coat	MAE Water	1.2–8.3				[133]
	MAE Ethanol 50–100%	1.1–52.9				[133]
	Acetone 70% (0.5% Acetic acid)	95.5–152.4	12.1–22.0		0.2–0.3	[136]
	MeOH 80%	5.2–21.6	2.7–20.9			[135]
Raw	MeOH 50%	3.7	1.3			[41]
	Acetone 70% (0.5% Acetic acid)	~6.5				[137]
Cooked	MeOH 50%	10.9–16.1	1.7–2.7			[41]
	Acetone 70% (0.5% Acetic acid)	~1.5–3.0				[137]
**Black beans**						
Whole seed	MeOH 70%	3.3	1.0	1.5		[130]
	EtOH 70–100%	3.2	1.2–1.3	0.4–1.6		[130]
	Acetone 50–80%	5.0–5.5	2.5–3.0	3.4–5.3		[130]
	Acetone 70% (0.5% Acetic acid)	5.9–12.8	3.2–7.1	5.7–8.6		[130,138]
	MeOH 60% (0.3–0.5% HCl)	0.9–24.0	~1.6	0.7–75		[131,139]
Cotyledon	MAE Water	3.8–5.5				[133]
	MAE Ethanol 50–100%	1.2–8.5				[133]
	Acetone 70% (0.5% Acetic acid)	1.8–2.5	0.20–0.50		nd	[136]
Seed coat	MAE Water	5.4–45.4				[133]
	MAE Ethanol 50–100%	1.1–60.8				[133]
	Acetone 70% (0.5% Acetic acid)	78.3–218.0	6.3–26.3		0.4–9.7	[136]
	SCF Ethanol 50%	66.6			7.3	[140]
	MeOH 80%	8.7–23.7	2.9–9.1			[135]
Raw	MeOH 50%	~5.0–7.0	~2.5–4.0		~1.5–2.5	[53]
	Acetone 70% (0.5% Acetic acid)	~7.5				[137]
Cooked	MeOH 50%	~1.2–6.0	~2.0–3.5	~54.6	~0.8–2.0	[53,141]
	Acetone 70% (0.5% Acetic acid)	~2.0–3.0				[137]
Digested	MeOH (0.5% HCl)	~0.2	~1.3	~8		[139]
**Red/violet beans**						
Whole seed	MeOH 70%	2.3	0.9	0.3		[130]
	MeOH (0.5% HCl)	~1.3	~2.0	~55		[139]
	EtOH 70–100%	1.2–2.5	1.2	0.1–0.5		[130]
	Acetone 50–80%	5.0–5.2	2.0–2.7	3.9–5.5		[130]
	Acetone 70% (0.5% Acetic acid)	5.9–9.18	2.9–5.8	5.4–8.4		[130,138]
Cotyledon	MAE Water	3.5–7.3				[133]
	MAE Ethanol 50–100%	1.1–10.9				[133]
	UAE Ethanol ~50%	~8.0–15.0	-			[134]
	Acetone 70% (0.5% Acetic acid)	2.2	0.32		nd	[136]
Seed coat	MAE Water	1.8–46.0				[133]
	MAE Ethanol 50–100%	1.0–70.6				[133]
	UAE Ethanol 42%/Ethyl acetate	~180–260	15.7	1.0–2.1		[134]
	MeOH 80%	16.5–58.0	4.3–36.4			[135]
	Acetone 70% (0.5% Acetic acid)	126.5	22.7		0.5	[136]
Raw	Acetone	~2.5–3.5	~1.2–2.0		~0.4–0.6	[53]
Cooked	Acetone	~1.5–2.5	~0.8–1.5		~0.2–0.4	[53]
Digested	MeOH (0.5% HCl)	~0.6	~2.0	~6		[139]
**Pink beans**						
Cotyledon	MAE Water	3.9–6.7				[133]
	MAE Ethanol 50–100%	1.0–8.9				
Seed coat	MAE Water	6.1–46.1				
	MAE Ethanol 50–100%	1.1–56.2				
**Speckled beans**						
Whole seed	Acetone 70% (0.5% Acetic acid)	2.4–5.2	2.7–2.8	3.0–5.0		[130]
Raw	Acetone	~3.0–4.5	~1.5–2.5		~0.5–0.8	[53]
Cooked	Acetone	~2.0–3.5	~1.0–2.0		~0.3–0.6	[53]
Seed coat	MeOH 80%	4.9–48.8	1.0–44.8			[135]
**Common beans**						
Whole seed	EtOH 70% (0.5% Formic acid)	~1.7–4.6	~1.0–2.5		~0.5–3.1	[142]
Raw	Water	3.5–5.0		1.0–1.9		[143]
	MeOH 50%	2.4	0.1			[41]
Cooked	Water	2.0–2.5		nd		[143]
	MeOH 50%	8.0–11.84	0.02–0.7			[41]

## Data Availability

No new data were created or analyzed in this study. Data sharing is not applicable.

## Abbreviations

The following abbreviations are used in this manuscript:

4E-BP1	Eukaryotic translation initiation factor 4E-binding protein 1
ACE	Angiotensin-converting enzyme
ACF	Aberrant crypt foci
ADP	Adenosine 5′-diphosphate
Akt	Protein kinase B
AMPK	AMP-activated protein kinase
Ang1	Angiopoietin 1
AOM	Azoxymethane
ATP	Adenosine triphosphate
Bax	Bcl-2 associated X-protein
BTK	Bruton tyrosine kinase
C3G	Cyanidin-3-glucoside equivalents
CB	Cooked beans
CD80	Cluster of differentiation 80
CGA	Chlorogenic Acid
cGMP	Cyclic guanosine monophosphate
COX	Cyclooxygenase
CVD	Cardiovascular disease
DPPH	2,2-diphenyl-1-picrylhydrazyl
EAM	Experimental autoimmune myocarditis
eNOS	Endothelial nitric oxide synthase
ERK1/2	Extracellular signal-related kinases 1 and 2
FAO	Food and Agriculture Organization
FOXO	Forkhead box O
GAE	Gallic Acid Equivalents
H_2_O_2_	Hydrogen peroxide
HDAC3	Histone deacetylase 3
HIV	Human immunodeficiency virus
IBD	Inflammatory bowel disease
IC_50_	Half maximal inhibitory concentration
ICAM-1	Intercellular adhesion molecule-1
IFN-γ	Interferon gamma
IGF-1	Insulin-like growth factor 1
IL	Interleukin
LC-TOF-MS	Liquid chromatography–time-of-flight mass spectrometry
LDL	Low-density lipoprotein
LOX	Lipoxygenase
LPS	Lipopolysaccharide
MAPK	Mitogen-activated protein kinase
MDM2	Mouse double minute 2 homolog
MDR	Multidrug resistance
METS	Metabolic syndrome
MMP	Matrix metalloproteinase
mTOR	Mammalian target of rapamycin
MyD88	Myeloid differentiation primary response 88
NDF	Non-digestible fraction
NF-κB	Nuclear factor kappa B
Ninj1	Ninjurin 1
NO	Nitric oxide
NOS	Nitric oxide synthase
Nrf2	Nuclear factor erythroid 2-related factor 2
PD	Peyronie’s disease
PE	Polysaccharide extract
PGE-2	Prostaglandin E2
PI3K	Phosphoinositide 3-kinase
PKG	Protein kinase G
PPAR-α	Peroxisome proliferator-activated receptor-α
PPT	Polyp Prevention Trial
ROS	Reactive oxygen species
SCFA	Short-chain fatty acids
Sirt1	Sirtuin 1
Src	Proto-oncogene tyrosine-protein kinase Src
Syk	Spleen tyrosine kinase
TAG	Triacylglycerol
TLR4	Toll-like receptor 4
TNF-α	Tumor necrosis factor alpha
VCAM-1	Vascular cell adhesion molecule 1
VEGF	Vascular endothelial growth factor
VLDL	Very-low-density lipoprotein
WHO	World Health Organization
WMN	Weimaining
αAI	α-Amylase inhibitor
